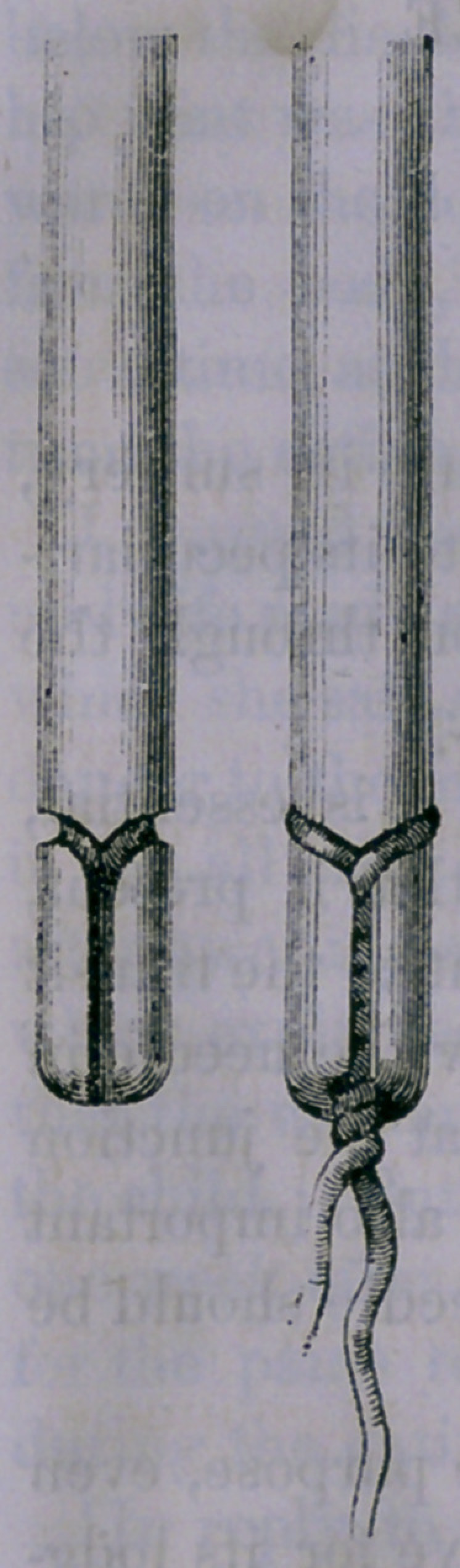# Needle for Wire Suture

**Published:** 1860-01

**Authors:** R. J. Levis

**Affiliations:** Surgeon to the Philadelphia Hospital


ARTICLE 7.

### NEEDLE FOR WIRE SUTURE.

BY R. J. LEVIS, M. D.,

Surgeon to the Philadelphia Hospital.

The present general use of the metallic suture in surgery, will make acceptable a form of needle adapted to its peculiarities, and which, by facilitating its introduction through the tissues, will add to its convenience and efficiency.

For the free passage of the needle and wire, it is essential, first, that the wire be securely held; second, that it present, at its connection with the needle, no impediment to the transit through the tissues; third, that it should follow the needle in a direct line, not allowing an angle to form at the junction which will require traction to overcome. It is also important for the convenience of the operator, that the needle should be readily threaded.

In using the ordinary surgical needle for the purpose, even though the needle be deeply grooved at the eye for its lodgment, the wire forms a ring-like attachment with the needle, which impedes its passage. A sort of hinge joint is also formed at the junction, which will be movable, no matter how tightly the wire be twisted, and there will be continually forming an angle with the needle, which is a great impediment to its use in delicate tissues, and in regions difficult of access, as the vagina, rectum, and fauces. Another inconvenience which has occurred with me in the use of the ordinary needle and the silver wire, is the liability of the wire to be pinched off by the forceps necessary to its introduction in the above localities.

These objections have induced some surgeons to introduce the wire by first passing the ordinary silk thread to which the wire is attached, and then drawn through.

For the purpose of overcoming these inconveniences in the ordinary use of the wire suture, I have devised a modification of the needle, which is so well illustrated by the accompanying proportionally collossal representations, as to render description almost unnecessary.

Its peculiarity is in what is usually the eye of a needle, though this is really without an eye, the attachment of the wire being accomplished by grooves in which it rests.

A groove deep enough for the lodgment of the wire, encircles the needle obliquely near its extremity, and leads into another groove which is vertical. The vertical groove is just wide enough at its entrance to admit the introduction of one wire at a time, but the inside of the groove being large enough to accommodate two wires, when both are introduced and twisted together, they are securely held. An attachment is thus effected which is as firm as if the needle and wire formed one continuous piece, and the wire being entirely encased within the grooves, it will traverse any tissue of the body without the least impediment.

There is a decided advantage in having the wire double for an inch or more following the needle, as any break in the wire invariably occurs very near to the needle.

This form of needle has been so extensively used in this city, as to thoroughly test its efficiency, and it has been preferred by the instrument makers, on account of its simplicity and the facility and cheapness with which it can be made.

Such needles, of sizes adapted to all uses, may be had from Mr. Gemrig, of Eighth street, or Mr. Kolbe, of Ninth street, in this city.—*Hospital Reporter*.

---

MERCURIAL STOMATITES.—Geo. T. Barker, D. D. S., in the *Dental Cosmos*, observes: "My mode of treating mercurial sore mouth comprises the systemic and local; for the first I can highly recommend the potassac chloras of the pharmacopœia; for the latter, freely scarify the gums, painting them, after each operation, with iodine."

## Figures and Tables

**Figure f1:**